# Are the detection and dimensions of peri‐implant buccal bone defects influenced by different implant materials and their location? An experimental cone‐beam computed tomography study

**DOI:** 10.1002/jper.70076

**Published:** 2026-01-30

**Authors:** Yulan Wang, Matheus L. Oliveira, Dorothea Dagassan‐Berndt, Michelle Simonek, Sebastian Kühl, Michael M. Bornstein

**Affiliations:** ^1^ State Key Laboratory of Oral & Maxillofacial Reconstruction and Regeneration, Key Laboratory of Oral Biomedicine Ministry of Education, Hubei Key Laboratory of Stomatology School & Hospital of Stomatology, Wuhan University Hubei China; ^2^ Department of Research University Center for Dental Medicine Basel UZB, University of Basel Basel Switzerland; ^3^ Division of Oral Radiology, Department of Oral Diagnosis Piracicaba Dental School, University of Campinas Piracicaba São Paulo Brazil; ^4^ Dental Imaging University Center for Dental Medicine Basel UZB, University of Basel Basel Switzerland; ^5^ Department of Oral Surgery University Center for Dental Medicine Basel UZB, University of Basel Basel Switzerland; ^6^ Department of Oral Health & Medicine University Center for Dental Medicine Basel UZB, University of Basel Basel Switzerland

**Keywords:** cone‐beam computed tomography, dental implant, exomass, metal artefacts, peri‐implant defect

## Abstract

**Background:**

The aim of this study is to evaluate the impact of different implant materials and arrangements within the field of view (FOV) and exomass on the diagnostic accuracy and image quality of cone‐beam computed tomography (CBCT) for the detection of peri‐implant buccal bone defects of various sizes.

**Methods:**

A total of 120 CBCT scans with three different implant materials with identical diameter (titanium [Ti], titanium‐zirconium [TiZr], and zirconium dioxide [ZrO_2_]) and three surgically created peri‐implant defect sizes (no defect, small defect: 3 mm, large defect: 6 mm) using two CBCT scanners (X800 and Planmeca) were generated. Three independent examiners assessed the scans for the presence of peri‐implant bone defects, rated the impact of metal artefacts on diagnostic confidence (visual analog scale [VAS] 0–10), and measured the maximum height and width of the defect, when present. Diagnostic performance (sensitivity, specificity, accuracy, and area under the receiver operating characteristic curve) was calculated by a generalized linear mixed model. Artefact perception (VAS) and measurement error (height and width) were analyzed using analysis of variance, with *p* < 0.05 considered significant.

**Results:**

CBCT imaging demonstrated high overall diagnostic accuracy for detecting surgically created peri‐implant bone defects when implants were positioned within FOV and the exomass. Nevertheless, defect size (*p* < 0.001) and implant material (*p* = 0.019) did affect the diagnostic accuracy significantly. Smaller bone defects (79.4%) were associated with a reduced diagnostic accuracy compared with larger defects (99.1%) and non‐defect sites (95.3%). ZrO_2_ dental implants (85.4%) were associated with a reduced diagnostic accuracy compared with Ti (97.5%) and TiZr implants (92.6%).

**Conclusions:**

CBCT reliably detects peri‐implant bone defects, irrespective of the implant material or the presence in the exomass. While ZrO_2_ implants and smaller defects can reduce diagnostic accuracy, its overall performance remains robust, warranting further clinical validation.

**Plain language summary:**

This study explored how well cone‐beam computed tomography (CBCT), a 3D dental imaging technique, can detect bone loss around dental implants. The authors tested three commonly used implant materials—titanium (Ti), titanium–zirconium (TiZr), and zirconium dioxide (ZrO_2_)—and created small and large buccal bone defects around them in a controlled laboratory setting. They also tested the effect of implants outside the CBCT scanning area to mimic real‐world clinical situations. Three trained examiners reviewed 120 CBCT scans to judge whether a bone defect was present, how much the implant material affected image assessment, and how closely their measurements matched the true size of the defects. Overall, CBCT was highly reliable in identifying bone defects, especially when defects were large. However, very small buccal defects were harder to detect, and zirconia implants produced more image distortion, making diagnosis slightly less accurate compared with Ti implants. Importantly, even when implants were positioned at the edge of the scan, CBCT still performed well. These findings support the use of CBCT as a dependable tool for evaluating bone loss around implants, while highlighting situations—such as small defects or zirconia implants—where interpretation should be made with extra care.

## 1 INTRODUCTION

Dental implants have become a standard method to replace missing teeth and to restore oral function as well as esthetics. However, with the widespread adoption of this treatment option, peri‐implantitis has also emerged as a clinically relevant complication. Peri‐implantitis is a type of peri‐implant disease, with a relatively high incidence rate, ranging from 15.15% to 69.5% at the patient level and from 11.65% to 60.4% at the implant level.^1^, ^2^ According to an international consensus statement from 2017, peri‐implantitis is defined as an “inflammation in the peri‐implant mucosa and subsequent progressive loss of supporting bone.”^3^ If the respective progressive bone destruction is left untreated, further bone loss may occur, which might eventually lead to implant failure and loss.

The diagnosis of peri‐implant defects involves both clinical and radiographic assessments. While probing is used to evaluate soft tissue conditions, radiographic methods have been recommended for assessing peri‐implant bone levels and for detecting bone defects.^4^ To assess the true three‐dimensional (3D) nature of peri‐implant bone diseases, CBCT scanning has been discussed as a potential imaging modality.^5^, ^6^, ^7^, ^8^ Increasingly, studies have validated the feasibility and effectiveness of CBCT in detecting peri‐implant bone defects, especially for complex peri‐implant defects.^9^, ^10^, ^11^ The main drawback of CBCT is the presence of beam hardening artefacts caused by high‐density dental materials, including implant materials, metallic restorations, and endodontic fillings. These beam hardening artefacts—such as bright streaks, darkened areas, and darkened areas between metallic or dense objects—do not appropriately represent anatomical structures and can obscure important details, leading to misinterpretation of bone defects, which are critical for diagnosis and treatment planning.^12^

In previous studies, researchers have found that the material and implant number, and examiner experience can influence the diagnosis of peri‐implant bone defects.^8^, ^13^, ^14^, ^15^ Zirconia implants can cause significant artefacts and have been found to create more detrimental artefacts in CBCT images than Ti implants.^15^, ^16^ Additionally, a higher number of implants can decrease the accuracy of diagnosing peri‐implant bone defects.^14^ Researchers have found that using a small field of view (FOV) can reduce tissue exposure to x‐rays, decrease scattered radiation, and thereby enhance image contrast and quality.^13^ Furthermore, the use of small FOV CBCT scans is also recommended in dental clinical practice to minimize the radiation dose exposure to patients.^17^ However, studies have revealed that structures outside, but surrounding the FOV, known as the exomass, are also exposed to the primary x‐ray beam during CBCT scans and can also produce artefacts, causing volumetric distortions of high‐density objects within the FOV, thereby potentially affecting the accurate diagnosis of peri‐implant bone defects.^18^ Nevertheless, previous studies regarding the diagnosis of peri‐implant bone defects using CBCT imaging have focused on implants located within the FOV, without studies examining the contribution of implants in the exomass to beam hardening artefacts formation within the FOV and their subsequent impact on the diagnosis of peri‐implant bone defects. This knowledge gap is particularly significant in dental clinical practice, as patients often have implants or metallic restorations in different locations of the same jaw, which might be in the exomass, potentially influencing the accuracy of diagnosing peri‐implant bone defects within the FOV.

To address this knowledge gap, the present study aimed to evaluate the impact of the material and arrangement of dental implants within the FOV and exomass on the diagnostic accuracy and image quality of CBCT for the detection of peri‐implant bone defects of various sizes. The results of the present study, which used pig mandibles, aim to enhance our understanding of CBCT imaging outcomes for assessing peri‐implant defects. The null hypothesis of the present study is that implant materials and the arrangement within FOV and exomass, and the size of the peri‐implant defect have no significant effect on the accuracy of peri‐implant bone defect diagnosis and image quality.

## 2 MATERIALS AND METHODS

### 2.1 Study design and sample preparation

**FIGURE 1:**
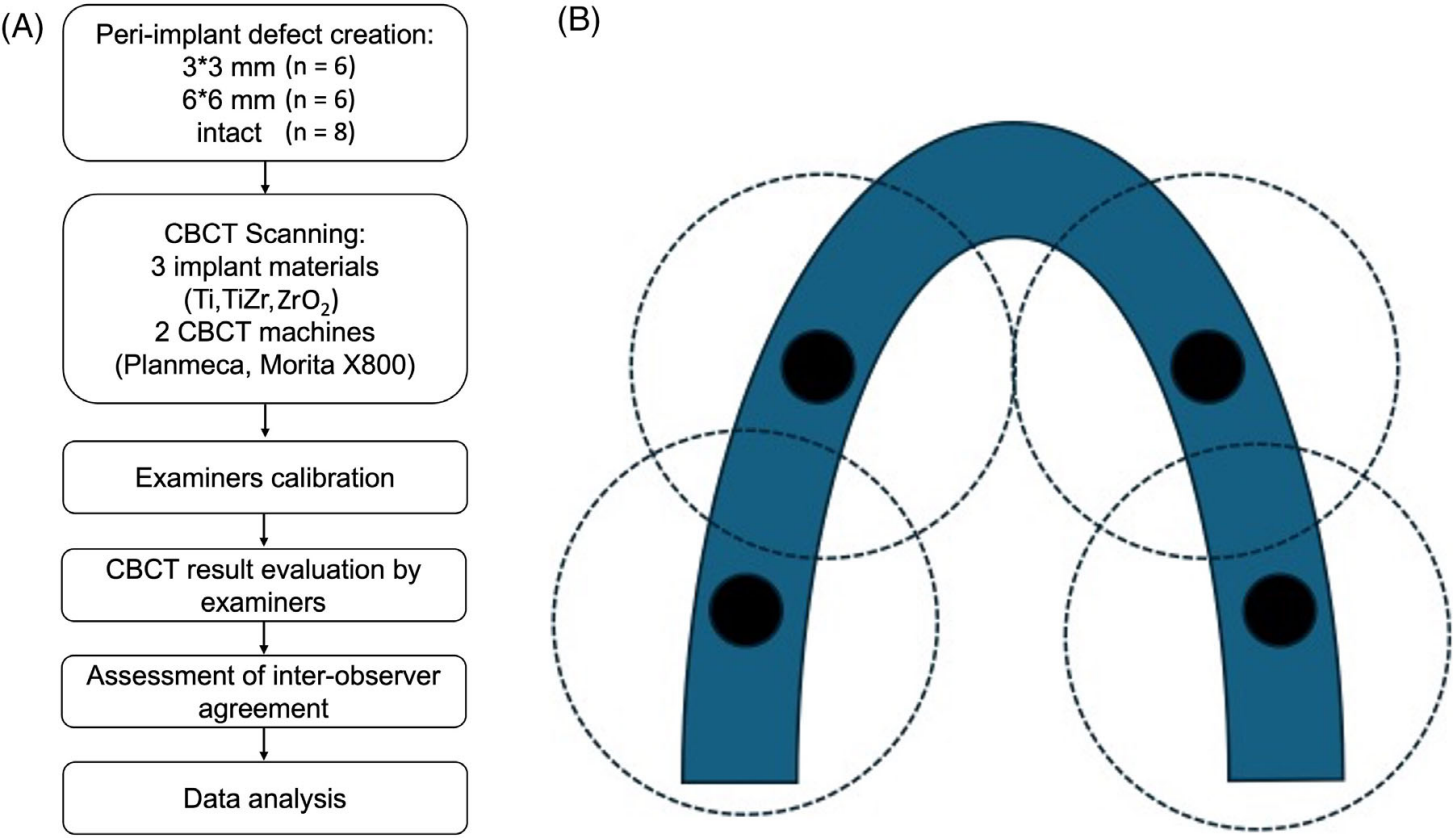
Schema illustrating the experimental design. (A) The flowchart of the study; (B) location of the fields of view (dotted empty circles; cone‐beam computed tomography [CBCT] scans centered on the respective dental implants (solid black circles), placed in the pig mandible (blue arch; anterior = premolar region/posterior = molar region).

Five fresh pig mandibles obtained from a slaughterhouse were used for this experimental study, following a previously published methodology.^8^ As no live animals were used, adherence to the ARRIVE guidelines was not applicable. Implant sites were prepared in the inferior cortex of the mandibles, with two implants placed in the premolar region and two implants placed in the molar region, corresponding anatomically to the upper border of the mandible where these teeth would normally be located (Figure 1).

**FIGURE 2:**
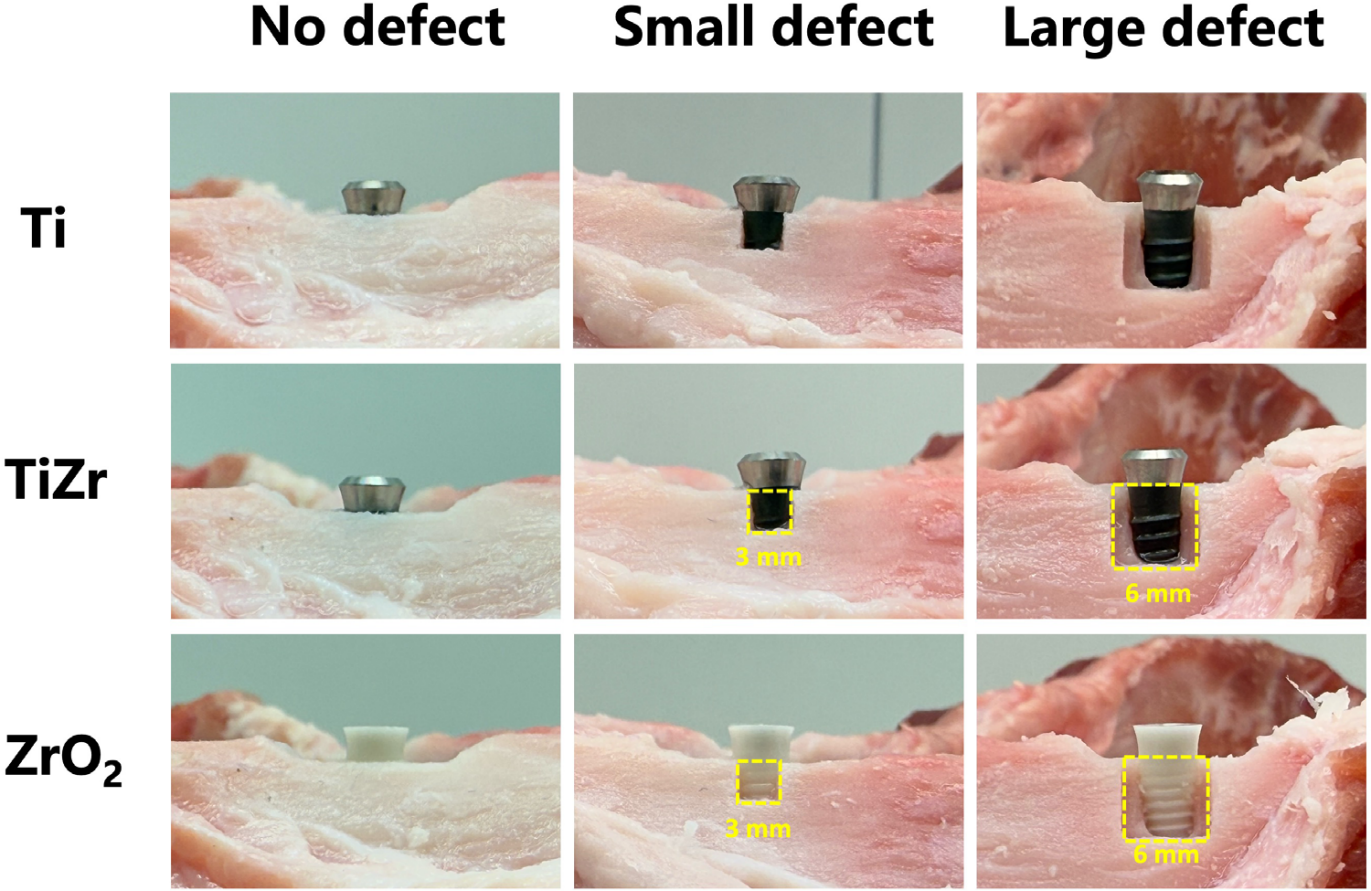
Clinical photographs of the buccal aspect of the pig mandibles highlighting two different implant materials (titanium [Ti] and zirconium dioxide [ZrO_2_]) and three peri‐implant conditions (no defect, small 3 × 3 defect, large 6 × 6 defect).

**FIGURE 3:**
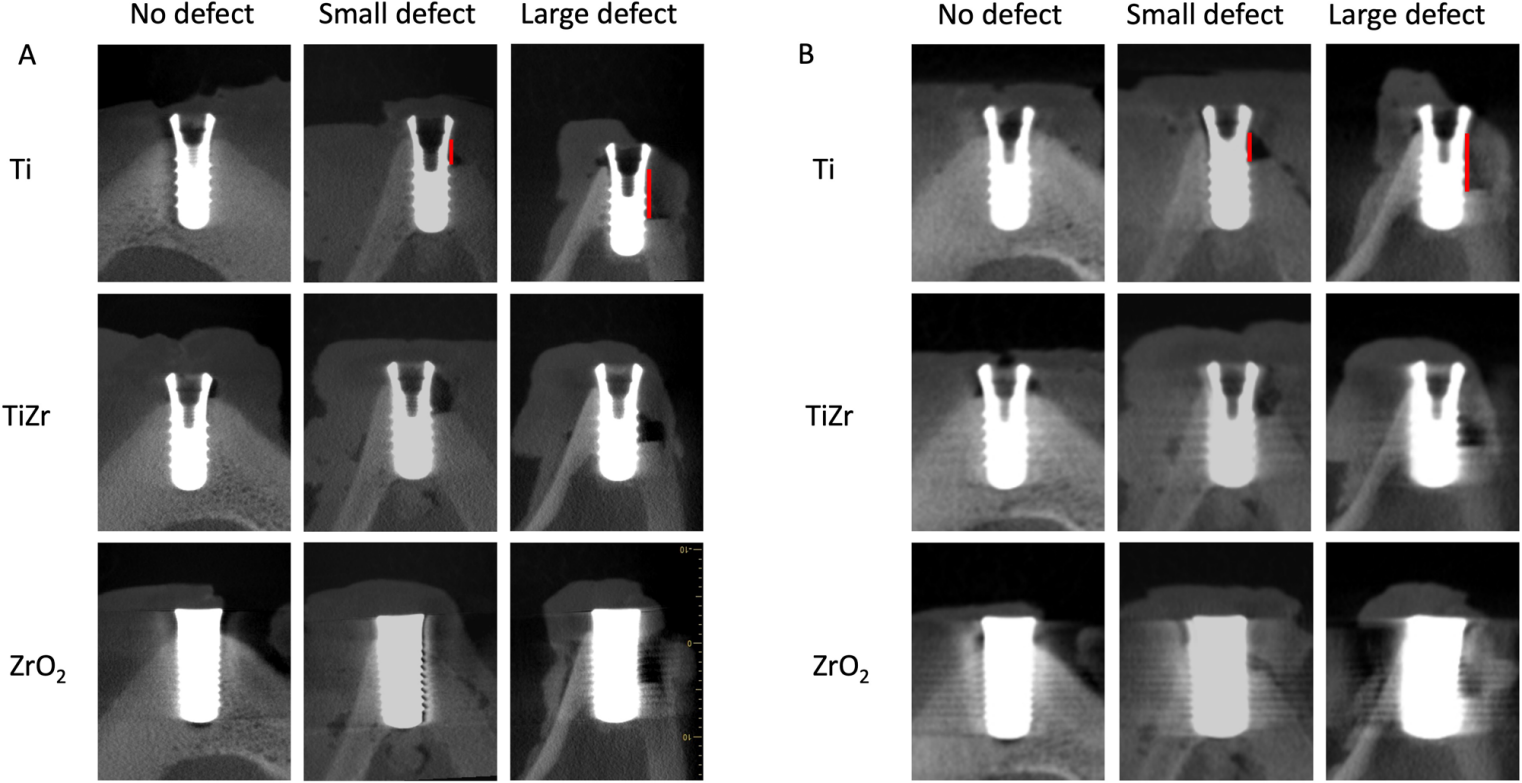
Representative cone‐beam computed tomography (CBCT) cross‐sectional images of the peri‐implant region by X800 (A) and ProMax 3D Max (B). Peri‐implant situation as seen from the buccal with three different implant materials (titanium [Ti], titanium‐zirconium [TiZr], and zirconium dioxide [ZrO_2_]) on the pig mandibles: left = no defect; middle = small defect (3 mm); and right = large defect (6 mm).

After elevation of flaps, four dental implants (specifications below) were inserted in premolar and molar regions on each side, following the manufacturer's standard implant placement protocols (Straumann Holding AG, Basel, Switzerland). A water‐cooled drilling device and round diamond burs were used to create peri‐implant defects. To ensure uniform peri‐implant defect morphology, all defects were standardized using a digital vernier caliper to measure the planned height and width. The initial defect outline was prepared with a round bur to approximate the target dimensions. Subsequently, a diamond polishing bur was used under continuous irrigation to refine the defect margins to achieve the predetermined size and to create a flat internal surface. The peri‐implant defects were randomly allocated by generating random numbers in Excel to ensure unbiased distribution, while intact implant sites served as controls (no defect; *n* = 8; Supplementary Material in the online *Journal of Periodontology*). Two types of buccal peri‐implant defects were created: a dehiscence‐type defect (small defect, *n* = 6) measuring 3 mm in width (mesial to distal bone distance), 3 mm in height (from the crestal bone to the bottom of the defect), and 1–2 mm in depth (from the vestibular implant surface to the outer oral bone edge), characterized by two parallel walls and a flat horizontal bottom at the most apical point (shown in Figures 2 and 3). The second dehiscence‐type defect (large defect, *n* = 6) had a larger size, measuring 6 mm in both width (mesial to distal bone distance) and height (crestal bone to the bottom of the defect), with 2–3 mm in depth (from the vestibular implant surface to outer oral bone edge), also featuring two parallel walls and a flat horizontal bottom at the most apical point. The 3 × 3 mm defect size was selected to simulate an early‐stage peri‐implant bone defect, closely approximating the clinically established 2 mm diagnostic threshold for peri‐implantitis.^19^ The 6 × 6 mm defect was included to represent an advanced bone defect with clear radiographic visibility.^20^ These clinically relevant sizes allow comprehensive assessment of CBCT performance across disease stages, particularly in artifact‐prone environments.

### 2.2 CBCT imaging settings

For the five pig mandibles, three types of dental implants were used: pure Ti implants (Straumann; Standard Plus Implant; dimensions of 4.1 × 10 mm), TiZr implants (Roxolid, Straumann Standard Plus Implant; dimensions of 4.1 × 10 mm), and ZrO_2_ dental implants (PURE Ceramic, Straumann; 4.1 × 10 mm in diameter and length). To ensure consistency across the study, the same five pig mandibles and the same four implants for each material were used across the study. To isolate the effect of implant material, implants were inserted and removed using standardized torque and positioning tools to maintain consistent angulation and depth. After each implant was inserted into the experimental site, the surrounding soft tissue was carefully repositioned to cover the implant and the bone defect prior to CBCT acquisition. Before and after implant replacement, the soft tissue was gently reflected and repositioned. The integrity of the bone defects was carefully checked with calipers after each implant insertion and removal to confirm that the defect dimensions remained unchanged. The pig mandibles were stored frozen and were thawed immediately before peri‐implant defect creation and CBCT scanning. Radiographic parameters were kept constant, and the exposure setup was rechecked using the CBCT system's built‐in alignment lasers after each replacement to ensure identical imaging geometry.

Two different CBCT scanners X800 (Morita, Kyoto, Japan) and ProMax 3D Max (Planmeca, Helsinki, Finland) were utilized. First, a large‐FOV scan (15 × 15 cm) was taken to ensure that all implants have been positioned at the same axial level (same height). Then, small‐FOV CBCT scans were performed centered on each implant, ensuring that the surrounding implants of the same pig mandible were located in the exomass (Figure 1). The following scanning parameters were used: (1) X800: FOV: 4 × 4 cm; voxel size:125 µm; standard mode: 100 kV, 5 mA, 360° rotation; exposure time: 17.9 s; (2) ProMax 3D Max: FOV: 5 × 5.5 cm; voxel size: 150 µm; standard mode: 90 kV, 5 mA, 200° rotation; exposure time: 15.1 s, with the metal artefact reduction (MAR) feature turned off. This resulted in a total of 120 CBCT scans (5 pig mandibles × 4 implant sites × 2 CBCT scanners × 3 implant materials).

### 2.3 Radiographic image assessment and outcome measurements

All CBCT scans were independently evaluated by three examiners with a background in dentomaxillofacial radiology (Master's or PhD students), who were blinded to the actual defect size. Assessments were performed in a low‐light environment using the OnDemand3D software (CyberMed Co., Seoul, South Korea) and a medical‐grade monitor (Barco MDRC‐2124; Barco, Kortrijk, Belgium). Prior to image analysis, the examiners underwent a calibration procedure on the evaluation protocol, supervised by an experienced dentomaxillofacial radiologist (MLO). To minimize the potential for visual fatigue, examiners were allowed to assess a maximum of 15 CBCT scans per session.

The following three parameters were assessed for all implants on the CBCT images by all three examiners:

1. Presence or absence of peri‐implant bone defects using a 5‐point scale, as follows: (1) definitely present, (2) possibly present, (3) uncertain, (4) possibly absent, and (5) definitely absent. Adjustments in brightness, contrast, and zoom setting were recommended.
2. The presence of peri‐implant artefacts and their impact on defect diagnosis were assessed using a 2‐point scale: “no” (VAS = 0) and “yes” (VAS 1–10). A digital visual analog scale (VAS) was used, consisting of a horizontal 10 cm line, with the left end marked as “0” indicating the absence of artefacts, and the right end marked as “10” indicating extremely severe artefacts. The examiners were instructed to mark a point along the line that best represented their subjective perception of artefact severity.
3. Linear measurements (in millimeters) of peri‐implant defect on the buccal aspect, when present, were made as follows: maximum height and maximum width.

Thirty days after the completion of image analysis, 20% of the CBCT volumes—randomly selected and equally distributed across the experimental conditions—were reassessed by all three examiners to evaluate intra‐examiner reproducibility.

### 2.4 Statistical analysis

Inter‐ and intra‐examiner reliability for the presence of peri‐implant defect was evaluated using the kappa statistic, while reliability for continuous measurements (VAS, defect height, and width) was assessed with the average‐measures intraclass correlation coefficient (ICC).

Diagnostic ratings were binarized: responses of 1 or 2 were classified as positive (defect present), while 4 or 5 were classified as negative (defect absent), while no responses of 3 was given by the examiners. A generalized linear mixed model with a binomial link function was employed to assess the effects of implant material, CBCT scanners, and defect size (as categorical fixed effects) on diagnostic accuracy, with sample ID and examiner included as random effects to account for repeated measurements. Sensitivity, specificity, accuracy, and area under the receiver operating characteristic curve (AUC) were calculated across implant materials, CBCT scanners, and peri‐implant defect sizes.

A linear mixed‐effects model (LMM) was used to analyze the effect of material on VAS scores, peri‐implant defect height and width, with examiner and sample ID included as random effects to account for repeated ratings and sample‐specific variability. After model fitting, residuals were examined using Q–Q plots, histograms, and the Shapiro–Wilk test to check model assumptions. Pairwise group comparisons were performed using Tukey's method based on estimated marginal means from the model.

All analyses were performed using R (version 4.5.1). Mixed‐effects models were fitted with lme4^21^ and lmerTest,^22^ and post‐hoc comparisons were performed using emmeans.^23^ Inter‐ and intra‐examiner reliability was evaluated with kappa and ICC using the irr^24^ and psych^25^ packages. ROC curves and AUC values were obtained via pROC,^26^ and figures were drawn using ggplot2.^27^ Statistical significance was set at *p* < 0.05. A *p*‐value < 0.05 was considered statistically significant (α = 0.05).

## 3 RESULTS

### 3.1 Intra‐ and inter‐observer consistency

For the five‐category assessment of peri‐implant defect presence, intra‐examiner consistency was high: Examiner 1 showed a Cohen's κ = 0.847, Examiner 2 had κ = 0.734, and Examiner 3 achieved κ = 0.780 (all *p* < 0.001). The inter‐examiner agreement among the three examiners was moderate for this variable, with a Fleiss’ κ of 0.401 (*z* = 5.73, *p* < 0.001).

For the examiner‐perceived peri‐implant artefacts and their diagnostic impact, as assessed by VAS scores, the intra‐examiner reliability was also high for all three examiners, with ICCs ranging from 0.80 to 0.91. The inter‐ examiner consistency was also excellent, with an average‐measure ICC of 0.84 (95% CI: 0.69–0.93, *p* < 0.001).

For the linear measurements of peri‐implant defect height and width, the intra‐examiner reliability was excellent for all examiners, with ICCs above 0.95. The inter‐examiner reliability was likewise excellent (0.96 for both height and width, 95% CI: 0.92–0.98).

### 3.2 Accuracy of peri‐implant defect detection

**FIGURE 4:**
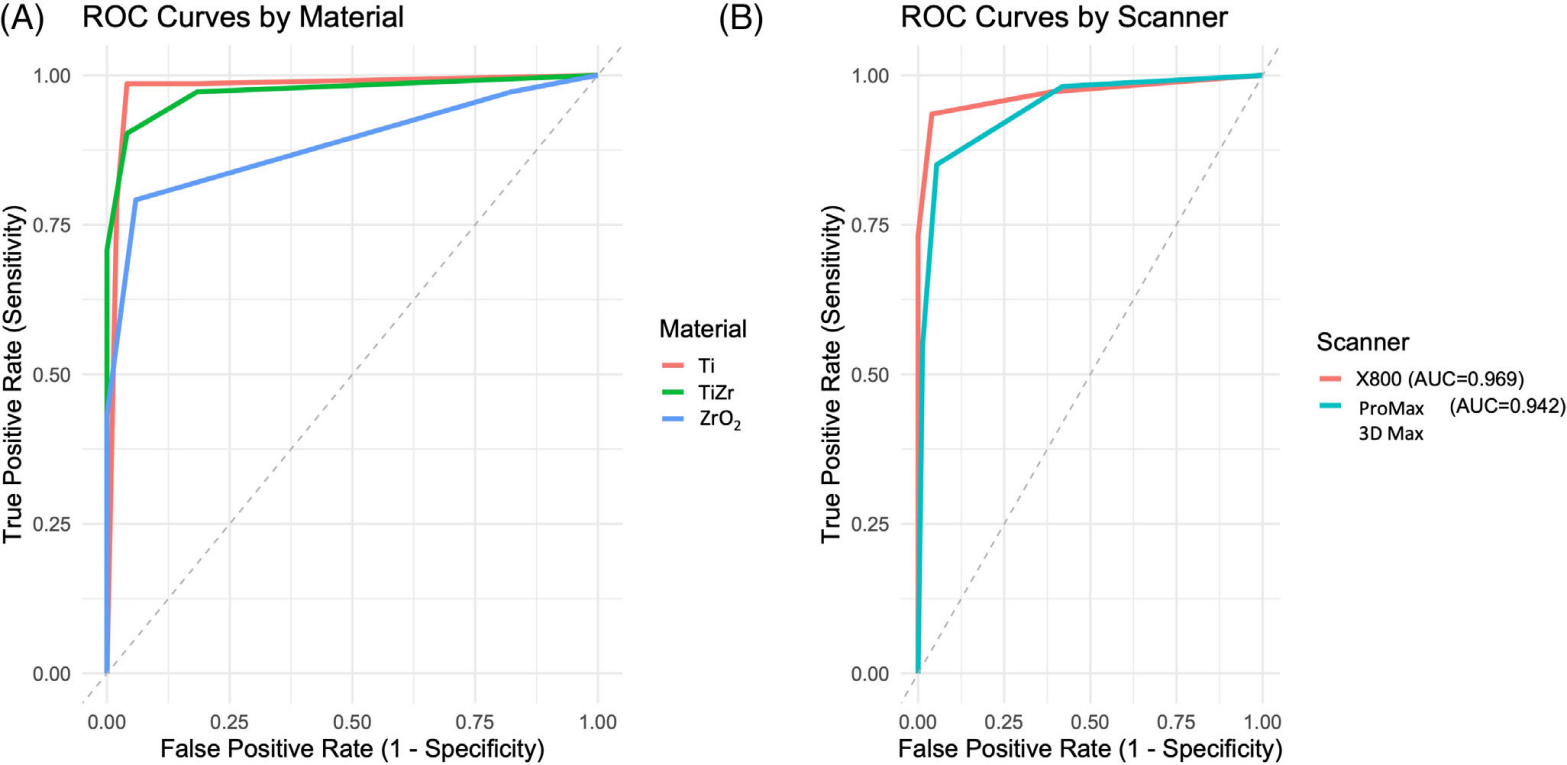
Receiver operating characteristic (ROC) curves for the diagnosis of peri‐implant defects, stratified by implant material and cone‐beam computed tomography (CBCT) scanner. (A) The ROC curves illustrate the diagnostic performance of observer majority voting for different implant materials (titanium [Ti], titanium‐zirconium [TiZr], and zirconium dioxide [ZrO_2_]). (B) The ROC curves illustrate the diagnostic performance of observer majority voting for different CBCT scanners (X800 and ProMax 3D Max). The true positive rate (sensitivity) is plotted against the false‐positive rate (1 – specificity) for each material and for each scanner.

Diagnostic accuracy differed significantly across the three implant materials (*p* = 0.019). Specifically, the ZrO_2_ implants were associated with significantly lower diagnostic accuracy compared with Ti implants (*p* = 0.018). As shown in Figure 4 and Table 1, Ti implants exhibited the highest diagnostic accuracy (97.5%), sensitivity (98.6%), and specificity (95.9%), with an AUC of 0.978 (95% CI: 0.949–1.000). TiZr also demonstrated high accuracy and AUC, while ZrO_2_ implants showed lower sensitivity (79.2%), accuracy (85.4%), and AUC (0.884).

**TABLE 1:** Sensitivity, specificity, and diagnostic accuracy for the detection of peri‐implant defects (yes/no).

Factor	Sensitivity	Specificity	Diagnostic accuracy	*p*	AUC (95% CI)
**Material**					
Titanium	0.986	0.959	0.975	**0.019(** * **)**	0.978 (0.949–1.000)
Titanium‐zirconium	0.903	0.959	0.926		0.972 (0.946–0.997)
Zirconia	0.792	0.941	0.854		0.884 (0.830–0.938)
**Scanner**					
X800	0.935	0.960	0.945	0.081	0.969 (0.945–0.992)
ProMax 3D Max	0.850	0.946	0.900		0.942 (0.910–0.974)
**Defect size**					
No defect	NA	0.953	0.953	**< 0.001(** * **)**	NULL
Small defect	0.794	NA	0.794		NULL
Large defect	0.991	NA	0.991		NULL

*Note*: Tukey's post‐hoc test: titanium versus zirconia: *p* = 0.018 (*); no defect versus small defect: *p* = 0.0057 (*); small defect versus large defect: *p* = 0.0038 (*).

Abbreviations: AUC, area under the curve; NA, not applicable.

* = significant.

For CBCT scanners, diagnostic accuracy showed no significant difference using the X800 unit (94.5%, AUC 0.969) and ProMax 3D Max (90.0%, AUC 0.942). Defect size had a significant effect on diagnostic accuracy (*p* < 0.001). Large defects showed the highest sensitivity and accuracy (both 99.1%), while small defects yielded lower sensitivity and accuracy (both 79.4%). For the “No defect” category, specificity and accuracy were both 95.3%.

### 3.3 The presence of peri‐implant artefacts and their impact on defect diagnosis shown by VAS

The impact of artefacts on diagnostic performance, assessed by mean VAS scores (0–10), was assessed by LMM to investigate the influence of materials, defect sizes, and CBCT devices. The results showed that all three fixed effects had a significant impact on VAS scores (Scanner: *p* = 0.0002; Material: *p* < 0.0001; and Defect category: *p* < 0.0001). Specifically, ZrO_2_ (7.44 ± 1.67) yielded significantly higher VAS compared with both Ti (2.01 ± 1.30) and TiZr (2.60 ± 1.89). X800 resulted in significantly lower scores compared with Planmeca. Random effects indicated substantial variability between examiners (SD = 1.23) and moderate variability among samples (SD = 0.86). The residual variance was 3.62, suggesting additional unexplained variability. Tukey‐adjusted pairwise comparisons further confirmed that ZrO_2_ had significantly higher ratings than Ti or TiZr, and that small defects (5.05 ± 3.00) led to higher VAS than large defects (3.21 ± 2.43).

### 3.4 Linear measurement of peri‐implant defects

For the measurement of defect height and width, the average underestimation was < 1 mm. However, in the presence of implants both within the FOV and exomass, a more pronounced underestimation was observed, with consistent differences between implant materials (ZrO_2_ vs. Ti and TiZr) and defect size (3 × 3 vs. 6 × 6 mm). As shown in Table 2, for height error, the LMM model showed significant main effects of material (*p* = 0.0035), defect size (*p* < 0.001), and a marginal effect of CBCT device (*p* = 0.051). In addition, the interaction between material and defect size was significant (*p* = 0.0017), suggesting that the material type influenced measurement bias differently across defect sizes. Post‐hoc comparisons indicated that ZrO_2_ specimens with smaller defects were significantly underestimated compared with Ti (*p* < 0.001). Overall, TiZr and ZrO_2_ groups tended to show lower mean height estimates (negative bias), whereas Ti exhibited a slight overestimation. The random‐effect variance was mainly attributed to sample ID (σ^2^ = 0.159), with residual variance of 0.537, indicating moderate within‐sample variability.

**TABLE 2:** Analysis of potential influencing factors on the impact of artefacts on linear measurements and assessment of peri‐implant defects.

Factor	Impact of artefacts on diagnostics (mean VAS ± SD, 0–10)	Height error (mm, mean ± SD)	Width error (mm, mean ± SD)
**Material**			
Titanium	2.01 ± 1.30	0.01 ± 0.43	−0.21 ± 0.42
Titanium‐zirconium	2.60 ± 1.89	−0.18 ± 0.67	−0.24 ± 0.68
Zirconia	7.44 ± 1.67	−0.4 ± 0.86	−0.44 ± 0.99
LMM	***p* < 0.001(** * **)**	***p* = 0.003542(** * **)**	*p* = 0.097623
**Scanner**			
X800	3.53 ± 2.74	−0.10 ± 0.46	−0.17 ± 0.48
ProMax 3D Max	4.51 ± 3.06	−0.28 ± 0.86	−0.43 ± 0.92
LMM	***p* < 0.001(** * **)**	*p* = 0.050795	***p* = 0.008626(** * **)**
**Defect size**			
No defect	3.85 ± 3.05	0.12 ± 0.33	0.13 ± 0.37
Small defect	5.05 ± 3.00	−0.38 ± 1.06	−0.63 ± 0.98
Large defect	3.21 ± 2.43	−0.42 ± 0.33	−0.54 ± 0.54
LMM	***p* < 0.001(** * **)**	***p* < 0.001(** * **)**	***p* < 0.001(** * **)**

*Note*: Tukey's post‐hoc test for VAS: titanium versus zirconia: *p* < 0.001 (*); titanium‐zirconium versus zirconia: *p* < 0.001 (*); no defect versus small defect: < 0.001 (*); small defect versus large defect: *p* < 0.001 (*). Tukey's post‐hoc test for height error: titanium versus zirconia: *p* = 0.0024; no defect versus small defect: *p* < 0.001 (*); no defect versus large defect: *p* < 0.001 (*). Tukey's post‐hoc test for width error: no defect versus small defect: *p* < 0.001 (*); no defect versus large defect: *p* < 0.001 (*).

Abbreviations: NA, not applicable; LMM, linear mixed model; VAS, visual analog scale.

* = significant.

For width error, significant effects were found for defect size (*p* < 0.001) and CBCT device (*p* = 0.009. The material and defect size interaction were not significant, but the defect size × CBCT device reached significance (*p* = 0.022), suggesting that scanner performance differed by defect size.

## 4 DISCUSSION

The results of this study partially reject the null hypothesis, indicating that implant material within the FOV and exomass as well as defect size, can influence image quality and diagnostic accuracy. To the best of our knowledge, this study is the first to explore the effect of dental implants with different materials placed within the FOV and exomass on the detection of peri‐implant bone defects with varying sizes. While there are significant artefacts from dental implants within the FOV and exomass in CBCT images, the technique still maintains high sensitivity and specificity for detecting peri‐implant defects. However, localized artefacts cause an increase in the perception of artefact on the examiner, especially with ZrO_2_ implants and small defects, and a slight underestimation in linear measurements was observed, especially with ZrO_2_ implants, consistent with the literature.^8^, ^15^ Given the increasing use of ZrO_2_ implants, the findings suggest that clinicians should interpret peri‐implant bone levels in CBCT scans with caution, especially for small buccal defects and when multiple implants are present. Adjusting FOV and MAR settings may partially mitigate diagnostic challenges. These insights emphasize the need for further optimization of scanning protocols and post‐processing techniques, which could be integrated with advancements in artefacts correction to enhance the diagnostic capabilities of CBCT scans.

Previous studies have explored the impact of artefacts, particularly from implants, placed within the FOV of a CBCT scan. Shokri et al. concluded that ZrO_2_ implants produce significantly more CBCT artefacts than Ti implants, which can be partially mitigated by adjusting voxel size and FOV.^28^ Schriber et al. found that artefacts generated by ZrO_2_ implants positioned within the FOV significantly reduced diagnostic accuracy.^8^ In clinical reality, however, patients often have additional implants located around the region of interest, and artefacts arising from these exomass implants may further complicate the diagnostic process. Oliveira et al. found when implants are located in the exomass of a small FOV, ZrO_2_ implants result in more pronounced artefacts, leading to a greater reduction of image quality, increased image noise and inhomogeneity of grayscale values.^18^ In addition to implant material and exomass, previous studies have shown that zirconia–zirconia implant–abutment interfaces and the presence of adjacent implants in the FOV have been reported to exacerbate artefacts and to reduce diagnostic accuracy.^29^, ^30^ These factors were not assessed in the present study to focus on incorporating implants within the FOV and exomass to simulate clinically relevant artefact conditions in a controlled manner. Despite the localized artefacts introduced by exomass implants, our results indicate that the diagnostic accuracy for detecting peri‐implant defects remained high, even for ZrO_2_ implants.

Another novelty of our study was the inclusion of different peri‐implant defect sizes. Specifically, we introduced a small defect of 3 mm, representing the initial stage of a peri‐implant bone defect, and a larger defect of 6 mm, indicative of a more advanced bone defect. The variation in defect sizes reflects typical scenarios in daily clinical practice. The study demonstrated that small buccal bone defects significantly decreased the diagnostic accuracy compared with larger defects or no defects. Moreover, a greater impact on examiners’ diagnostic confidence was found when small defects were present, as reflected in higher VAS scores. Previous studies have shown that the thickness of the buccal bone plate can also influence CBCT diagnostic accuracy, with thinner plates significantly reducing defect detectability.^29^, ^30^ However, due to the inherent variability of the porcine specimens, the thickness of the buccal bone plate cannot be fully standardized, and comparisons across predetermined thickness categories could not be performed.

Candemil et al. reported that current MAR algorithms were largely ineffective in correcting such artefacts. With the recent advancements in artificial intelligence and image post‐processing techniques, several methods have been proposed to mitigate beam hardening artefacts in CBCT imaging.^31^ For instance, Oliveira et al. developed and applied a denoising diffusion probabilistic model‐based deep learning model to reduce exomass‐related artefacts in CBCT images, achieving significantly improved image quality^18^; Hyun et al. reported deep learning‐based artefact reduction methods using intraoral scans to enhance image quality and diagnostic accuracy^32^; Park et al. introduced a neural‐representation‐based reconstruction technique that effectively minimizes metal artefacts in dental CBCT imaging.^33^ These studies highlight the ongoing progress in artefact reduction techniques and emphasize the importance of integrating advanced metal artefact correction algorithms and optimized low‐dose scanning protocols in the future.

This study employed an *ex vivo* model using pig mandibles, although they are commonly used in dental imaging research,^34^, ^35^ their clinical translatability requires clarification. Although, the pig mandible shares a comparable cortical–trabecular bone structure and is visualized similarly in CBCT scans like human jaws, this model has limitations such as the inability to fully replicate human anatomy (soft‐tissue thickness and bone density) and lacks patient movement, which may influence artefact expression and diagnostic accuracy. Thus, while the model provides a controlled platform for comparing implant materials and exomass effects, translation to human clinical conditions should be made with caution.

Furthermore, buccal peri‐implant bone defects were chosen because they are both the most prevalent peri‐defect type and represent the most diagnostically challenging. As a result, the findings may not be generalizable to defects located on the mesial or distal aspects or with a circular, crater‐like morphology. The square‐shaped lesions were standardized to allow for reproducible preparation and quantitative measurement of defect dimensions. Although the contour does not perfectly mimic the naturally rounded morphology of peri‐implant bone defect, such standardized geometry ensures comparable radiodensity transitions across sites and minimizes confounding variability due to irregular borders. Finally, while earlier studies have extensively evaluated the influence of material type and implant within FOV, the combined presence of both FOV and exomass implants—reflecting the typical coexistence of adjacent metallic structures in clinical practice—has not been systematically addressed. The present setup, therefore, represents an important intermediate step toward understanding how exomass artifacts interact with in‐FOV implant imaging, extending previous work into a more clinically realistic context. An additional limitation is the exclusive focus on dental implants. Future studies should incorporate more anatomically realistic bone models, vary implant positions relative to the FOV, include irregular or patient‐derived defect morphologies, and restorative variations including crowns, bridges, and endodontic posts to further validate and extend the clinical applicability of the present findings.

## 5 CONCLUSIONS

CBCT imaging can reliably detect peri‐implant bone defects regardless of dental implant material type or their location within the FOV and exomass. Although diagnostic accuracy decreases with the presence of zirconia implants and small peri‐implant bone defects, overall performance remains high. Nevertheless, interpretation of peri‐implant tissue around zirconia implants depicted in CBCT images should be done cautiously, especially when assessing early peri‐implant bone loss.

## AUTHOR CONTRIBUTIONS

**Yulan Wang**: Contributed to experiments; data analysis, and manuscript drafting. **Matheus L. Oliveira**: Contributed to study conception; experiments, and revised the manuscript. **Dorothea Dagassan‐Berndt**: Contributed to CBCT detection methodology and manuscript revision. **Michelle Simonek**: Contributed to model establishment and manuscript revision. **Sebastian Kühl**: Contributed to model establishment and manuscript revision. **Michael M. Bornstein**: Contributed to study conception and critical revision of the manuscript. All authors approved the final version.

## CONFLICT OF INTEREST STATEMENT

The authors declare no conflicts of interest.

## Supporting information



Supporting Information
