# Supplementary material for: Are the detection and dimensions of peri‐implant buccal bone defects influenced by different implant materials and their location? An experimental cone‐beam computed tomography study
Source: J Periodontol. 2026 Jan 30;97(9):1860–70. doi: 10.1002/jper.70076 (PMC13569053; doi:10.1002/jper.70076)
Supplement: Supplementary file 1 [file JPER-97-1860-s001.docx]

Table 1. Distribution of dental implants with peri-implant bone defects in the pig mandibles.

|  | Left premolar | Right premolar | Left molar | Right molar |
| --- | --- | --- | --- | --- |
| Pig mandible 1 | No defect | Small defect | Large defect | No defect |
| Pig mandible 2 | Large defect | No defect | Small defect | Large defect |
| Pig mandible 3 | Small defect | Small defect | No defect | Large defect |
| Pig mandible 4 | Large defect | No defect | No defect | Small defect |
| Pig mandible 5 | No defect | Large defect | Small defect | No defect |

No defect: Intact / no peri-implant defect (n=8)

Small defect: Peri-implant defect size = 3 × 3 mm (n=6)

Large defect: Peri-implant defect size = 6 × 6 mm (n=6)

Table 2. Reliability coefficients for peri-implant outcome measurements.

| **Variable** | **Inter-examiner Reliability** | **Examiner 1**  **Intra-examiner** | **Examiner 2**  **Intra-examiner** | **Examiner 3 Intra-examiner** |
| --- | --- | --- | --- | --- |
| Presence of peri-implant defect | 0.401 (-) | 0.847 (-) | 0.734 (-) | 0.780 (-) |
| Presence of peri-implant artefacts and their impact on defect diagnosis | 0.84 (0.69- 0.93) | 0.69 (0.40 - 0.85) | 0.66（0.36-0.84） | 0.83 (0.65 - 0.92) |
| Height of peri-implant defect | 0.96 (0.92 - 0.98) | 0.95 (0.90 - 0.98) | 0.91 (0.81 - 0.96) | 0.93 (0.84 - 0.97) |
| Width of peri-implant defect | 0.96 (0.92 - 0.98) | 0.98 (0.94 - 0.99) | 0.92 (0.82 - 0.96) | 0.91 (0.80 - 0.96) |

Fleiss’ κ (inter-examiner) and Cohen’s κ (intra-examiner) was used for the categorical variable. Continuous-variable inter-examiner reliability is reported as ICC (2,k), and intra-examiner reliability for each examiner as ICC (3,1). All reliability estimates include 95% confidence intervals and achieved p < 0.001.
